# Outcomes of Conservatively Managed Patients With Abdominal Aortic Aneurysm: A Single-Centre Experience

**DOI:** 10.7759/cureus.74391

**Published:** 2024-11-25

**Authors:** Muhammad Zulfiquar, Haytham Hamid, Tamer El-Sayed

**Affiliations:** 1 Vascular Surgery, Sunderland Royal Hospital, Sunderland, GBR; 2 Vascular Surgery, South Tyneside and Sunderland National Health Service (NHS) Foundation Trust, Sunderland, GBR

**Keywords:** abdominal aortic aneurysm, comorbidities, conservative management, ruptured abdominal aortic aneurysm, survival rates

## Abstract

Introduction: The results of patients at one hospital who were judged eligible for conservative care of abdominal aortic aneurysms (AAA) are examined in this research. Optimizing patient care and management tactics requires an understanding of the mortality trends and causes of death within this group.

Methodology: Sunderland Royal Hospital carried out a single-center retrospective analysis between May 2018 and January 2024. Out of the 447 individuals who had a AAA diagnosis, 396 had repairs, while 51 were rejected. Medical records were used to gather information on demographics, aneurysm features, length of survival, and reasons for death. The reasons for death and survival rates were ascertained using statistical analysis.

Results: Thirty (58.8%) of the 51 patients had passed away, while 21 (41.1%) were still alive. The remaining 26 fatalities (87%) were ascribed to various causes, such as myocardial infarction (27%), cancer (17%), and chronic obstructive pulmonary disease (COPD) (13%), with just four deaths (13%) being caused by ruptured AAA (rAAA). According to the research, the one-year survival rate was 17.6%, and the first six months after choosing not to intervene were when the majority of deaths occurred.

Conclusion: Comorbidities have a substantial influence on outcomes for patients treated conservatively for AAA, as evidenced by the low proportion of rupture-related fatalities. This study emphasizes the need for more investigation, especially large-scale, population-based studies, to gain a better understanding of AAA patient care and outcomes.

## Introduction

A life-threatening illness known as abdominal aortic aneurysm (AAA) is defined by the abnormal dilatation of the abdominal aorta, which can burst and cause abrupt death if treatment is not received [1]. An AAA is usually diagnosed when the aortic diameter is greater than 3 cm, and it primarily affects older people, particularly males over 65 years. Historically, surgical intervention has been the mainstay of AAA care, mainly because aneurysms larger than 5.5 cm in diameter carry a risk of rupture [2,3]. However, depending on factors like age, comorbidities, or aneurysm features, not all patients who arrive with aneurysms that need surgery right away are good candidates for such an invasive operation [4,5]. For patients who do not fit the requirements for surgery or who are at high surgical risk, conservative management, which includes careful monitoring and lifestyle or medication changes, has therefore become a viable alternative [6-8].

The emphasis on conservative care has increased recently, particularly since improved imaging methods and risk assessment instruments allow physicians to track the advancement of AAA with greater precision. Even though conservative therapy is less intrusive, it is nevertheless crucial to comprehend its long-term effectiveness and related patient outcomes [9]. Limited research has been done on conservatively treated AAA patients, and the findings are frequently contradictory, with some studies showing positive outcomes and others pointing to an increased risk over time. This has led to the necessity for more research to ascertain the effectiveness and safety of this strategy, especially in particular patient populations.

Providing important insights into the outcomes of conservatively handled AAA patients who were judged unfit for surgery or who chose non-surgical care is the goal of this single-center study. This study aims to determine the survival rates, aneurysm progression, and incidence of adverse events by examining data from a targeted cohort. This will help to advance our understanding of the role that conservative therapy plays in treating AAA. In order to improve patient care and maximize clinical results, this research assists doctors in honing patient selection criteria for conservative therapy.

## Materials and methods

Study design and setting

Patients with AAA who were judged unsuitable for surgery were the subject of this single-center, retrospective observational research, which was carried out at Sunderland Royal Hospital. From May 2018 to January 2024, data was gathered, offering a thorough analysis of patient outcomes, comorbidities, and survival indicators.

Study population

The original evaluation included 447 patients with AAA, 396 of whom were receiving endovascular or open treatments. However, after evaluation by the multidisciplinary team (MDT) meeting of the vascular department, 51 patients (35 men and 16 women; median age of 82.5 years, age range 66-96 years) were not eligible for any kind of intervention. After being informed about the operation and its hazards, these individuals either decided against surgery or were judged unfit for intervention because of the substantial operating risks. After the decision against intervention was made, the vascular department did not regularly follow up with patients in this group.

Data collection

Records from the municipal council, general practitioner (GP), and hospital were used to compile patient data. Patient demographics, aneurysm features, comorbidities, survival status, and, if relevant, cause of death were among the factors gathered. When available, clinical letters and death certificates were used to confirm the cause of death for dead patients. To fill in any gaps in the cause-of-death information, local coroners, general practitioners, and city council records were consulted.

Statistical analysis

To examine patient demographics, comorbidities, and survival outcomes, descriptive statistics were computed. Patients' survival outcomes were produced in table form to show patterns within this group and survival analysis was performed to evaluate survival length after choosing against intervention. The statistical association between comorbidity load and survival length was also investigated for individuals with numerous comorbidities, offering information on how underlying medical issues affect patient outcomes.

Ethical considerations

The study complied with the Declaration of Helsinki's tenets, guaranteeing strict adherence to data protection and patient confidentiality procedures. Patient permission was not required because this study collected data retrospectively, and the data were anonymized to protect patient privacy. All data sources were also examined for authenticity and correctness, and extra care was taken to manage sensitive data-like cause-of-death records in accordance with national and institutional data protection laws.

## Results

Fifty-one individuals with an abdominal aortic aneurysm (AAA) diagnosis were examined in this single-center retrospective analysis. At the time of follow-up, 30 (58.8%) of them were dead, and 21 (41.1%) were still alive. There were 35 patients in the male population, 14 of whom were alive and 21 of whom were dead, while there were 16 patients in the female population, seven of whom were alive and nine of whom were dead (Table 1).

**Table 1:** Patient status overview rAAA: Ruptured abdominal aortic aneurysm.

Status	Total No. of Patients (n=51)	Count	Percentage
Overall	51		
Alive		21	41.1%
Deceased		30	58.8%
- Died of rAAA		4	13.3%
- Other causes		26	86.6%

Of the 496 AAA patients in the larger group, 51 patients (10.5%) were rejected for surgical repair. This group's median age was 82 years (IQR 77-88), and 67.3% of its members were men. The study's AAA patients' clinical and demographic features were reflected in the median AAA diameter of 60 mm (IQR 55-66).

A detailed analysis of the aneurysms' features was conducted. With a mean size of 60.01 mm, a median of 60 mm, and a mode of 55 mm and 57 mm, the aneurysms' sizes differed among the patients. Juxtarenal (8%) and thoracoabdominal (2%) were less prevalent than infrarenal, which accounted for 90% of all aneurysms (Table 2). In particular, 47 individuals had infrarenal, four had juxtarenal, and two had thoracoabdominal out of the 52 patients who were rejected for treatment.

**Table 2:** Aneurysm type distribution

Aneurysm Type	Count	Percentage
Infrarenal aneurysms	45	90
Juxta renal aneurysms	4	8
Thoracoabdominal aneurysms	2	2

Among patients treated conservatively, the size distribution of AAA varied from 47 to 91 mm, with the majority (22 patients) falling between 55.8 and 64.6 mm. With a median of 60 mm and typical sizes (modes) of 55 and 57 mm, the average AAA size was 60.01 mm. This suggests that the majority of patients had AAA, which measured about 60 mm, which would have affected the choice of conservative treatment. Only five patients had AAA larger than 73.4 mm, indicating that larger AAA was less prevalent (Table 3).

**Table 3:** Aneurysm size distribution

Size Range (mm)	Number of Patients
47-55.8	15
55.8-64.6	22
64.6-73.4	9
73.4-82.2	3
82.2-91	2
Mean	60.01
Median	60
Mode	55, 57

Of the 30 patients who passed away, 13.3% died solely from ruptured AAA (rAAA), while the remainder (86.6%) died from reasons other than rAAA. According to Table 4, the top three causes of death for individuals falling under the "other causes" category were myocardial infarction (four patients), chronic obstructive pulmonary disease (COPD) (four patients), and cancer (six patients). This highlights the fragility of the patient group by showing that chronic illnesses, rather than complications linked to AAA, were the primary cause of mortality in this cohort.

**Table 4:** Causes of death rAAA: Ruptured abdominal aortic aneurysm; COPD: Chronic obstructive pulmonary disease.

Cause of Death	Count
Total Deceased (n=30)	
- Died of rAAA	4
- Other causes	26
- Malignancy	6
- COPD	4
- Myocardial Infarction	4
- Pneumonia	3
- Frailty of age	4
- Pancreatitis	2
- B/L acute lower limb ischemia	1
- Sepsis of unknown origin	1
- End-stage vascular dementia	1

Over a range of time periods, the survival results of the living patients were evaluated (Table 5). The one-year rate among the 21 survivors was 17.6%, followed by the two-year rate of 15.6%, the three-year rate of 5.8%, and the four-year rate of 1.9%. Notably, none of the patients lived for more than five years.

**Table 5:** Survival rates of alive patients

Survival Duration	Number of Patients	Percentage
one-year	9	17.6%
Two-year	8	15.6%
Three-year	3	5.8%
Four-year	1	1.9%
Five-year	0	0%

On the other hand, the period of survival for the patients who passed away showed that 13.7% of them died after a year, and 29.4% died within six months (Table 6). According to this research, the first six months are when non-intervention patients are most at risk for death.

**Table 6:** Survival rates of deceased patients

Survival Duration	No. of Patients	Percentage
Died within six months	15	29.4%
Died after one year	7	13.7%
Died after two years	4	7.8%
Died after three years	2	3.9%
Died after four years	1	1.9%
Died after five years	1	1.9%

The kinds and sizes of AAA among the individuals who passed away from ruptured AAA varied greatly. A 62 mm suprarenal AAA, a 46 mm descending thoracic, a 75 mm infrarenal, a 71 mm infrarenal, and a 78 mm juxtarenal AAA were among the features of the aneurysms in these individuals. One patient died within the first month after the decision to forego surgery, but the other patients survived for four, 14, and 26 months, respectively (Table 7).

**Table 7:** Patients who died due to rAAA rAAA: Ruptured abdominal aortic aneurysm.

Patient No.	Aneurysm Type	Size	Duration of Survival
1	Thoracoabdominal aneurysm	62 mm suprarenal aneurysm + 46 mm descending thoracic aneurysm	37 days
2	Infrarenal aneurysm	75 mm	32 days
3	Infrarenal aneurysm	71 mm	One year, Two months
4	Juxtarenal aneurysm	78 mm	Two years, Four months

## Discussion

The study's conclusions shed important light on the demographic traits, prognoses, and causes of death of AAA patients, especially those receiving conservative treatment. The 51 patients that were observed had an overall survival rate of 41.1%, which is consistent with other studies showing that different survival rates depend on treatment and intervention techniques. For example, a related research with AAA patients found a median survival time of 14.1 months and an overall survival rate of 42.3%, indicating that survival results in non-intervention cohorts might be similar in various contexts [10].

Our cohort's median age of 82 years and preponderance of male patients (68.6%) are consistent with known trends in AAA demographics. Research continuously demonstrates that older guys are more likely to have AAA and that mortality increases with age [11]. Additionally, 90% of the AAA in our sample were infrarenal, which supports results from other research that show infrarenal AAA to be the most prevalent kind [12]. A mean size of 60.01 mm was found in the AAA size distribution, which is consistent with research that indicates a higher risk of rupture and death is associated with bigger AAA [13]. This highlights even more how crucial it is to regularly monitor and evaluate patients with AAA, particularly those who are thought to be unsuitable for surgery.

The study's survival results show that although some individuals can live for several years without surgery, after the first year, survival drastically drops. Just 17.6% of patients were still alive after a year, and by the fourth year, that number had fallen to 1.9%. This pattern is in line with research from previous studies that show the greatest risk of death happens in the first six months after diagnosis, especially for patients who choose conservative treatment [14]. The multifaceted character of patient outcomes is shown by the reasons for death found in this study. 86.6% of the deceased passed away from reasons unrelated to AAA, mostly chronic illnesses, including cancer and myocardial infarction. The significance of thorough geriatric evaluation and comorbidity management in the AAA patient group is highlighted by this observation [15]. Prior studies have highlighted the fact that AAA patients are frequently elderly and fragile, which leaves them vulnerable to a number of chronic illnesses that can impair their general health [16].

The results show a wide variety of AAA sizes and kinds among the individuals who specifically died from ruptured AAA. Certain patient traits or health states may contribute to prolonged longevity, even in the presence of substantial pathology, as evidenced by the data showing that some patients lived for lengthy periods of time despite having burst AAA. Given that different patients react differently to the same clinical situations, this lends credence to the idea that tailored patient care is essential [17].

Limitations and future suggestions

There are many restrictions on this study. First, the findings may not be as broadly applicable as they may be due to the small sample size of 51 individuals. More data would be available from a bigger cohort, which may also show other trends or results. Second, because this study was only carried out at one facility, bias might be introduced because of different management and patient selection procedures. Third, insufficient information on comorbidities, lack of postmortem examinations in most patients, and other pertinent variables that can affect patient outcomes may result from the retrospective design's reliance on already-existing medical records. To confirm these results and improve generalizability, multi-center studies with bigger sample numbers should be taken into account in future research. Furthermore, longitudinal research on the long-term results of AAA patients-especially those treated conservatively-would shed more light on survival patterns and quality of life after diagnosis. In order to improve overall patient outcomes and survival rates, it is also crucial to implement standardized methods for the assessment and management of comorbidities in AAA patients. Lastly, more research into how screening programs contribute to the early identification of AAA may result in enhanced management tactics and higher survival rates for high-risk groups.

## Conclusions

Only 13% of the patients who passed away had ruptured abdominal aortic aneurysms (rAAA) according to this audit. The remaining 87% of deaths were due to reasons other than AAA, mainly myocardial infarction, malignancy, COPD, and sepsis. The results imply that the underreporting of rAAA's occurrence may be a result of misdiagnosis. All things considered, this single-center study emphasizes how crucial it is to manage comorbidities in AAA patients, suggesting that more research is required to fully comprehend these consequences, ideally through large population-based prospective studies.
